# Sub-County Life Expectancy: A Tool to Improve Community Health and Advance Health Equity

**DOI:** 10.5888/pcd15.170187

**Published:** 2018-01-25

**Authors:** Vickie L. Boothe, Leslie A. Fierro, Amy Laurent, Margaret Shih

**Affiliations:** 1 Centers for Disease Control and Prevention, Atlanta, Georgia; 2 Claremont Graduate University, Los Angeles, California; 3 Seattle and King County Public Health, Seattle, Washington; 4 Los Angeles County Department of Public Health, Los Angeles, California

## Abstract

Compared with people in other developed countries, Americans live shorter lives, have more disease and disability, and lag on most population health measures. Recent research suggests that this poor comparative performance is primarily driven by profound local place-based disparities. Several initiatives successfully used sub-county life expectancy estimates to identify geographic disparities, generate widespread interest, and catalyze multisector actions. To explore the feasibility of scaling these efforts, the Centers for Disease Control and Prevention and the Council of State and Territorial Epidemiologists initiated a multiphase project — the Sub-County Assessment of Life Expectancy. Phase I participants reviewed the literature, assessed and identified appropriate tools, calculated locally relevant estimates, and developed methodological guidance. Phase I results suggest that most state and local health departments will be able to calculate actionable sub-county life expectancy estimates despite varying resources, expertise, and population sizes, densities, and geographies. To accelerate widespread scaling, we describe several successful case examples, identify user-friendly validated tools, and provide practical tips that resulted from lessons learned.

## Need for Sub-County Population Health Indicators

Safer workplaces, vaccinations, improved motor-vehicle safety, and other twentieth-century public health achievements measurably improved health and increased longevity worldwide (1). In the United States, life expectancy at birth (LE), a key population health measure, increased steadily, reaching an all-time high of 78.8 years in 2012 (2). Since then, however, American LE has stalled. After increasing modestly from 2012 to 2014, LE unexpectedly declined to 78.8 years in 2015, adding to concerns about our nation’s health (2,3). Despite spending more than double on health care than other developed countries, Americans increasingly live shorter lives, experience more disease and disability across the lifespan, and lag on most population health measures (4).

Profound and persistent local geographic disparities are primary drivers of America’s poor performance (5,6). In 2010, LEs for females in Marin County, California (85.02 y), and males in Fairfax County, Virginia (81.67 y), were equivalent to the longest-lived countries of Japan and Switzerland. In contrast, LEs for males in McDowell County, West Virginia (63.90 y), and females in Perry County, Kentucky (72.65 y), were lower than estimates for Bangladesh and Nicaragua (5). Researchers suggest that these disparities are driven by several factors, including health care access; socioeconomic factors; and environmental, behavioral, and physiological risk factors (5).

Addressing America’s poor performance requires a shift in approach, which has focused historically on medical interventions, behaviors, and lifestyle choices (4). Accordingly, public health officials have called for development of locally relevant and timely neighborhood-level health and other indicators to drive actions that address underlying health determinants such as housing, economic development, and environment (7). This latest call to action adds to the growing body of literature documenting an urgent need for community-level health indicators. Without valid, reliable local indicators, health departments are constrained in their ability to detect disparity “hot spots,” identify correlated determinants, and catalyze effective, targeted, multisector actions (8–11).

## Advantages of Life Expectancy at Birth Compared with Other Local Measures

Unique mathematical and other properties suggest that local LE is better suited for driving actions than are other mortality measures (12). LE enables direct comparisons across time and geographic areas with diverse population structures and is easier to interpret than standardized mortality ratios or age-adjusted mortality rates (13–15). Stratifying LE by demographic characteristics such as race and income can elucidate disparities, inform resource allocation, and catalyze policy changes (9). LE has greater utility than modeled-based small-area estimates of national and state health survey data, which cannot be used to evaluate intervention effects (16) and can be affected by recall and selection bias (17). Furthermore, several studies document the feasibility of generating robust and accurate LE for small populations. Using Monte Carlo simulations, researchers evaluated methods for generating LE for the United Kingdom’s electoral wards, which in 2001 had a mean population of 5,959 (range, 995–35,770) (11,13,18). The adjusted Chiang II life table method was judged to produce accurate and reliable estimates for populations of 5,000 person-years-at-risk or more with standard errors of approximately 2 years. Because ward-level LE disparities were estimated to exceed 10 years, standard errors of 2 years and associated 95% confidence intervals of approximately 7 years allowed identification of wards with statistically different values (18). Subsequently, researchers evaluating methods for local jurisdictions in New South Wales and in Austria, Italy, Japan, Spain, Sweden, and the United Kingdom confirmed that populations smaller than 5,000 person-years-at-risk yielded biased LEs with standard errors too large for meaningful analysis (15,19). Other documented sources of LE bias include contextual factors such as large nursing home populations, which skew distribution of local population structures (15,19).

These findings hold promise for generating local LEs for most American populations. With average population sizes of 4,000 and a general range of 1,200 to 8,000 (20), census tracts are similar in size to United Kingdom wards. Also, US Census data on nursing home populations and other group quarters is readily available (21).

## Demonstrated Utility of Local Life Expectancy at Birth

Government agencies in England, Wales, Greece, and Australian New South Wales have used local LE for many public health applications, including identifying and tracking measurable reductions in health disparities (11,22), evaluating intervention effectiveness (14), and planning and funding local health services (14). Local LE has also been used to explore contributions of socioeconomic and environmental conditions to population health. For example, researchers exploring LE disparities in England and Wales reported the most important determinant to be “material poverty,” which is further influenced by sociodemographics, housing quality, and local economic policies (23,24).

Maps of LE help drive actions. Mapped LE inequalities between England’s northern and southern local authorities generated widespread media interest and catalyzed creation of Health Equity North, a collaboration of northern councils, the volunteer sector, National Health Service (NHS), and academia. Subsequent independent inquiry into root causes spurred national policy changes and increased community-centered investments focused on economic growth to reduce poverty; early childhood investments; transfer of authority and resources to local governments; and NHS services expanded beyond health care to address social determinants such as poverty, housing, education, and unemployment (25). For example, NHS partnered with Public Health England and others to fund 10 Healthy New Towns pilot sites, where 200,000 new housing units constructed in health-promoting neighborhoods will be monitored and evaluated for health effects (26).

In the United States, LE maps catalyzed local initiatives by highlighting disparities of up to 25 years across nearby neighborhoods in metropolitan areas including Oakland, California; Chicago, Illinois; Los Angeles, California; and New Orleans, Louisiana (9,10,27,28). Case examples from the Los Angeles County Department of Public Health (LACDPH) and Public Health–Seattle & King County (PHSKC) provide additional evidence of the utility of local LE maps.

### Life expectancy at birth in Los Angeles County

In 2009, LACDPH examined LE disparities in the county. Although LE had increased steadily since 1991, large disparities were observed, including a nearly 18-year difference between black males (69.4 years) and Asian/Pacific Islander females (86.9 years). LACDPH recognized that actions addressing the underlying social and environmental health determinants were needed to reduce these disparities and advance equity. Partnerships with cities and unincorporated communities were established, and maps examining LE at matching geographic levels were created to increase engagement.

The adjusted Chiang II method was used to calculate single-year LE for 103 cities and unincorporated communities with populations greater than 15,000 (11,29). The Economic Hardship Index (EHI) was used to examine the relationship between LE and community-level social and economic conditions across communities (29). The EHI is a composite of 6 indicators (crowded housing, poverty, unemployment, educational attainment, population dependency, and income level) that provides a more complete picture of neighborhood conditions than any individual measure. The strong inverse relationship between the EHI score and LE prompted LACDPH to publish a report that ranks cities and communities by LE and economic hardship that was broadly disseminated via press releases and in print and electronic form to city mayors, council members, planners, and representatives from other health-related sectors such as education, housing, transportation, and business (27).

The report received substantial coverage in local, national, and international media and on local websites and blogs. Resulting increased awareness of the connection between social issues and health led to reframed city and community discussions around root causes of health and increased community engagement and motivation to act. For example, the report provided justification for a 2015 formal amendment to the Los Angeles General Plan, elevating health as a priority for the city’s future expansion and development. The amended plan includes a policy vision and measureable objectives for creating healthier communities through increased affordable housing, cleaner environments, and safer neighborhoods (30). Finally, the report strengthened LACDPH’s engagement with city and community leaders, education, business, and other nonhealth sectors and raised awareness of the importance of a Health-in-All-Policies approach, which considers the health implications of non-health–sector policies (31).

### Life expectancy at birth in Seattle–King County

**Figure 1:**
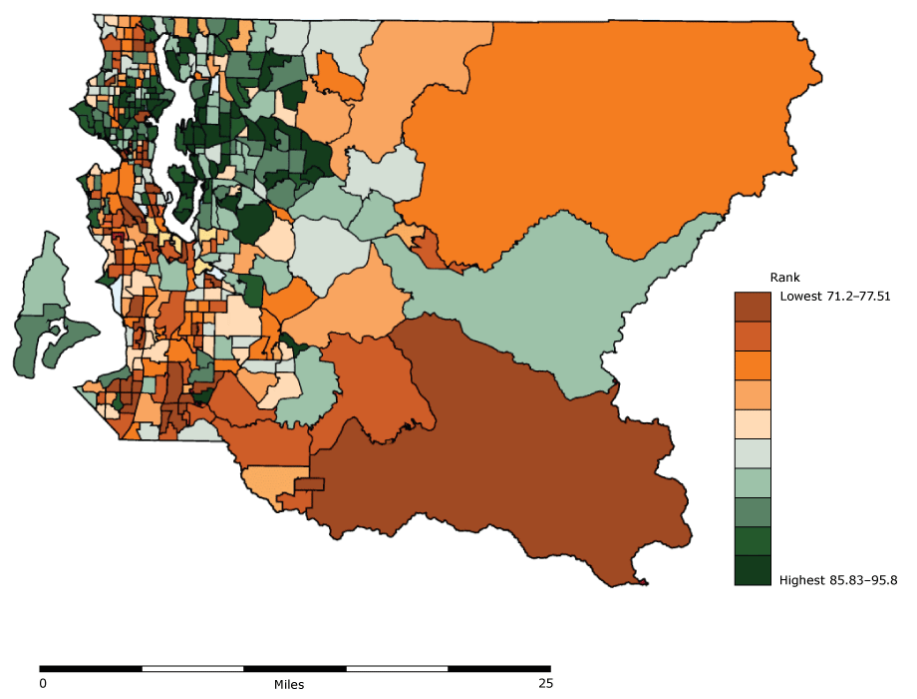
Estimated life expectancy at birth by census tract in King County, Washington, based on 2008–2012 mortality data.

PHSKC staff calculated LE for King County using the adjusted Chiang II method (13) and 2012 mortality data. LE in King County (81.2 years) was substantially longer than LE in the United States. However, pronounced disparities across race/ethnicity and subregions were evident, so PHSKC staff examined census tract–level LE. In 2010, King County’s 398 tracts averaged 4,800 (range, 1,286–11,056) people. PHSKC used geocoded mortality data from 2008 through 2012 assigned to census tracts and locally generated population estimates to generate LE data. After suppressing cells with statistically unreliable estimates, results showed an LE gap of approximately 24 years between the shortest-lived and longest-lived tracts (Figure 1).

PHSKC then used a Bayesian hierarchical model to generate mapped small-area estimates of modifiable risk factors such as adult obesity, smoking, adverse childhood experiences, preventable hospitalizations, poor housing conditions, high unemployment, low income, and adult frequent mental distress (32). Strikingly similar spatial patterns of disparities in LE and risk factors led to identification of potential communities for engagement; catalyzed an ongoing partnership between PHSKC, the Department of Community and Human Services, Seattle Foundation, and Living Cities; and led to formation of the Communities of Opportunity (COO) (33). COO focuses on improving equity in communities through system-level and policy-level solutions led by or engaging the local community. The COO collective impact framework includes community-identified achievement goals with identified indicators to measure progress. Desired results are that all people thrive economically; have quality, affordable housing; are healthy; and are connected to the community. To date, more than 90 community residents and 45 community organizations and their leaders have codesigned solutions (33).

## Resources for Calculating Sub-County Life Expectancy at Birth

In September 2014, the Centers for Disease Control and Prevention (CDC) and the Council of State and Territorial Epidemiologists (CSTE) initiated the multiyear Sub-County Assessment of Life Expectancy (SCALE) project. The goal of SCALE Phase I, which ended in June 2015, was to identify appropriate methods for calculating actionable sub-county LE and develop easy-to-use resources designed to assist other health departments. For LE to be considered actionable, the method needed to produce accurate estimates for most of the jurisdiction’s populations with standard errors and confidence intervals narrow enough to permit identification of areas with significantly higher or significantly lower LE values.

Phase I participants included a CDC senior scientist, an external evaluator, and scientists from LACDPH and PHSKC, recruited on the basis of their previous experience. Additionally, scientists from 6 state health departments (Florida, Maine, Massachusetts, New York, Washington, and Wisconsin) were invited to participate because they varied in size and resources, their jurisdictions represented diverse geographies and populations, and they had experience examining relationships between small-area health and environmental indicators through an initiative of the National Environmental Public Health Tracking Network (EPHTN).

Phase I participant activities included a literature review to identify feasible methods, successful case studies, and gold-standard parameters. After each jurisdiction independently tested various approaches, a consensus was reached to adopt the adjusted Chiang II method and associated software developed by the South East Public Health Observatory (34). Phase I participants also developed a draft guidance document (Guide) clarifying methodological decision points (eg, age categories, addressing zero cells, minimum population sizes) and sharing lessons learned. SCALE Phase I and subsequent activities are described in Table 1.

**Table 1:** SCALE Phase I and Phase II Activities, United States, 2015–2017

SCALE Phase I (January 2015–May 2015)	SCALE Phase II (June 2015–June 2017)
Conducted a literature review to understand the approaches, available parameters, and lessons learned from previous efforts associated with constructing small-area LE estimates.	Recruited and oriented new states/localities to methods and general project purpose/approach.
Reviewed common approaches used in the literature for calculating direct small-area LE estimates and arriving at initial decisions about methods.	New state/localities pilot tested draft materials from Phase I and provided feedback through the evaluation.
Identified other existing tools for calculating LE that might easily be adopted/adapted (SEPHO).	States/localities assessed potential refinements in methods to expand geographic coverage by performing several sensitivity analyses.
Compared calculations produced by SEPHO tool with other methods for generating LE estimates (SAS [SAS Institute, Inc] and STATA [Stata Corp, LP] code from previous LE efforts), and refined approach.	Implemented evaluation.
Developed an evaluation plan for Phase II.	Compiled lessons learned, refined tools and methodological recommendations, updated Guide and related resources, prioritized list of remaining issues and future actions.
Products included 1) drafted Guide for state/local health departments with SEPHO tool as approach used, 2) obtained sub-county estimates for Phase I states/localities, 3) held 2015 CSTE conference presentation, 4) made evaluation plan.	Products included 1) created SCALE website, 2) revised tools for estimating LE, 3) revised/updated Guide, 4) held 2016 and 2017 CSTE conference presentations, 5) evaluated findings 6) completed manuscripts.

Abbreviations: CSTE, Council of State and Territorial Epidemiologists; LE, life expectancy at birth; SCALE, Sub-County Assessment of Life Expectancy; SEPHO, South East Public Health Observatory.

The initial objective was standardized calculation of census-tract LE estimates using 5 years of death data (2008–2012) and 2010 census or local population estimates. Because SCALE is a user-driven initiative with a primary goal of supporting local actions, participants were encouraged to adapt the proposed objective and methods to meet their unique needs.

To evaluate feasibility of generating sub-county LE, interviews with each jurisdiction were conducted using questions designed to answer the following questions:

1. What resources are required for health departments with varying resources and diverse populations to calculate actionable sub-county LE for the majority of their jurisdiction?
2. What methodological and data challenges were encountered and how were they addressed?

## SCALE Phase 1 Results and Lessons Learned

All jurisdictions reported successful calculation of actionable LE for most sub-county areas in less than 1.5 years, with 7 of the 8 participating jurisdictions completing calculations in less than 1 year.

### Characteristics of participating jurisdictions and LE calculation approaches

Table 2 identifies characteristics of participating health departments and Table 3 describes the various LE approaches. Participating jurisdictions varied greatly on total expenditures, staffing, and total population size. Annual state health department expenditures for 2011 ranged from $108.08 million to $2.16 billion, and staffing ranged from 387 to 15,026 full-time equivalent employees (35). The 2016 Census population estimate for Maine of 1.3 million was smaller than those of the 2 county jurisdictions and approximately 16 times smaller than the estimated 20.6 million Florida residents (36).

**Table 2:** SCALE Jurisdiction Characteristics, United States, 2015–2017

Jurisdiction	Jurisdiction Characteristics				
	Total Expenditures^a^ in 2011, $	Workforce^a^ Full-Time Equivalents, 2011	Geographic Unit	Population Size^b^ in 2016, Millions	Population Per Square Mile^b^ in 2010
Florida Department of Health	2.16 billion	15,026	State	20.6	350.6
Los Angeles County Department of Public Health	NA	NA	County	10.2	87.4
Maine Department of Health and Human Services	108.08 million	387	State	1.3	43.1
Massachusetts Department of Public Health	762.57 million	2,933	State	6.8	839.4
New York State Department of Health	1.72 billion	3,127	State	19.8	411.2
Public Health–Seattle & King County	NA	NA	County	2.1	912.9
Washington State Department of Health	537.21 million	1,650	State	7.2	101.2
Wisconsin Department of Health Services	258.55 million	395	State	5.8	105.0

Abbreviations: NA, not available; SCALE, Sub-County Assessment of Life Expectancy.

a Source: Association of State and Territorial Health Officials (35).

b Source: US Census Bureau (36).

**Table 3:** Jurisdiction’s Life Expectancy Characteristics, United States, 2015–2017

Jurisdiction	Characteristics of Life Expectancies (Standard Error, 2 Years)				
	Basic Geographic Units	Number Years of Data	Minimum Population Size	Mean Population Size	Maximum Population Size
Florida Department of Health	Census tract	5	672	4,796	33,041
	Zip code	5	295	21,138	72,248
Los Angeles County Department of Public Health	Census tract	5	1,072	4,417	12,581
Maine Department of Health and Human Services	Minor civil division	10	1,012	4,213	64,504
Massachusetts Department of Public Health	Census tract	5	1,164	4,616	9,557
New York State Department of Health	Census tract	5	NA	NA	NA
Public Health– Seattle & King County	Census tract	5	1,280	5,248	10,776
Washington State Department of Health	Census tract	5	1,112	4,795	13,201
Wisconsin Department of Health Services	Zip code	10	540	7,488	60,953

Abbreviation: NA, not available.

Florida, Massachusetts, New York State, Washington, and PHSKC successfully calculated census tract–level LE with standard errors of less than 2 years for most of their populations using 5 years of data. All but one met the recommended minimum population size of 1,000 residents achieving 5,000 person-years-at-risk. Florida results included census tracts with the smallest and largest populations, 672 and 33,041 residents, respectively. Smaller and sparser Maine populations (37) required 10 years of data to generate LE with acceptable standard errors for most of their populations at the Minor Civil Divisions (MCDs) level. MCD is a US census bureau term for primary governmental divisions of a county such as townships. Wisconsin also required 10 years of data to calculate actionable LE at the zip code level. Aggregating data over time increases precision; however, the resulting LE may not reflect current conditions and increases the risk of numerator and denominator mismatch, which can bias standard errors (11,13,18). LACDPH chose to calculate single-year LE for areas with populations greater than 15,000.

### Data challenges

Population and mortality data were readily available; however, some data sets were unsuitable or required additional manipulation. Florida explored the feasibility of calculating LE for inter-census periods, using American Community Survey (ACS) data. ACS data lacked population counts for the ideal age-intervals for calculating LE (<1 year and 1–4 year categories vs 0–4 years) and had high margins of error at the census tract level. As a result, LE estimates generated by using ACS data varied substantially from LE estimates generated by using 2010 Census data for the same sub-county area (46 years vs 65 years, respectively).

Erroneous and missing mortality data in some jurisdictions increased time and resource requirements. Special record requests were often necessary for residents dying in neighboring state jurisdictions. In Maine, mortality data lacked addresses before 2011; therefore, town of residence was used to assign deaths to MCDs. In New York State, mortality records required geocoding using varied batch and iterative techniques. Hospital records were used to correct incomplete or inaccurate address information. Geographic imputation techniques using age, race/ethnicity, town, and zip code were used to geocode remaining cases with missing addresses. Ultimately, census tracts were assigned to 99.97% of mortality records. However, these labor-intensive methods extended the project by several months. An article describing the New York State methods is under development.

### Small number issues

Each jurisdiction included areas with populations too small to meet the recommended 5,000 person-years-at-risk. Florida, Massachusetts, PHSKC, Washington, and Wisconsin suppressed all LE values with standard errors greater than 2. Florida also suppressed improbable LE values of less than 66 years. The percentage of suppressed sub-county areas ranged from 3% to approximately 15%.

Before calculating LE, New York State excluded 18 census tracts that had no people and consisted of bodies of water, airports, and an uninhabited island. After exploratory analyses, tracts where more than 50% of the population lived in group quarters were also excluded. Consistent with the effects of nursing homes on LE values (16), improbable LE values were generated for tracts with large prison, military base, or college populations. Approximately 2.6% census tracts were ultimately excluded. Additionally, a New York State Geographic Aggregation Tool (38) was used to aggregate several neighboring census tracts until all had a minimum of 60 deaths and standard errors of less than 2 years.

Maine conducted exploratory analyses examining the effect of using a minimum standard error of 2 versus a standard error of 3 years, minimum number of deaths (>60), and minimum denominator (5,000 person-years). Depending on the rule(s), between 28% (standard error <3) and 46% (standard error <2 or deaths >60) of MCDs needed to be suppressed. Ultimately, Maine aggregated 10 years of data and several adjacent areas using the Geographic Aggregation Tool (38) until LE for all MCDs had a standard error of less than 2 years.

## Discussion

America’s lagging health status and persistent local disparities warrant bold actions that address all determinants of health, including social and environmental factors. Identifying and quantifying local disparities is a necessary first step for selecting, implementing, and documenting the impact of interventions (9,39). LACDPH and PHSKC and other case studies document the use of sub-county LE for quantifying disparities and catalyzing multisector actions (26,31,33).

Many LE methodological challenges were identified, such as small number issues, missing and erroneous data, and lack of suitable population data for noncensus years. Small, sparse populations in 2 jurisdictions prohibited the calculation of census tract–level LE using 5 years of data. All jurisdictions included areas requiring suppression of LE data or additional temporal or geographical aggregation. However, these solutions may not be as effective for even smaller or more sparsely populated jurisdictions. Finally, LE does not fully reflect health status or other dimensions of well-being through the life course (40).

**Figure 2:**
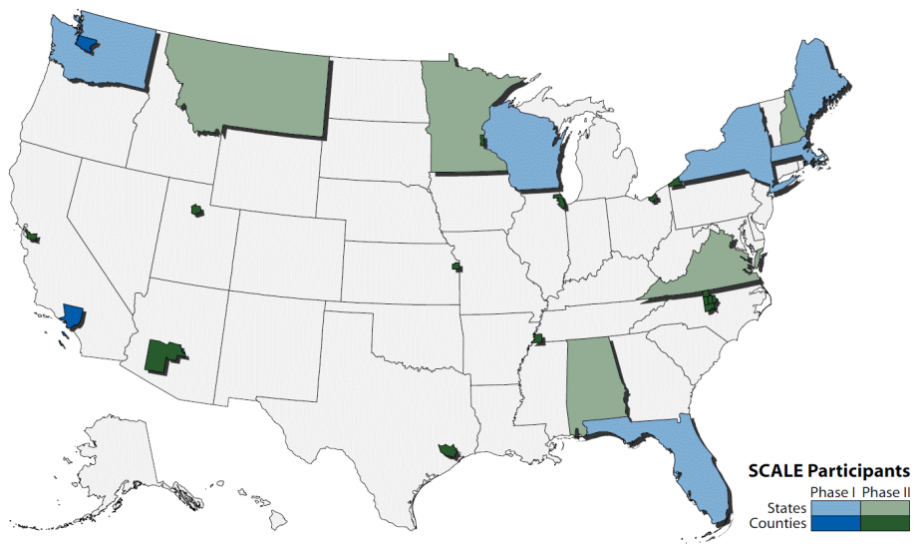
United States map identifying health department jurisdictions of SCALE Phase I and II participants. Abbreviation: SCALE, Sub-County Assessment of Life Expectancy.

As part of SCALE Phase II, initiated in June 2015, 17 additional health departments successfully calculated sub-county LE, pilot tested the Guide, and provided feedback on its usability and utility (Figure 2). In September 2016, CSTE launched a SCALE website (www.cste.org/page/SCALE/Sub-County-Assessment-of-Life-Expectancy-SCALE-Project.htm), which includes version 1.0 of the updated Guide and other user-friendly resources. A joint SCALE and EPHTN workshop was held in October 2016, with objectives of prioritizing future collective activities and supporting sub-county LE calculation by the 20 EPHTN grantees not previously engaged in SCALE.

Prioritized future activities include identifying key local social determinant and health indicators for co-release with LE estimates; assessing feasibility of generating summary population measures that better reflect overall health, including health-adjusted life expectancy; identifying LE visualization and messaging best practices; and evaluating the utility of local LE for monitoring and evaluating the health effects of local policies and programs.

Current and planned SCALE resources directly respond to calls for locally relevant data capable of identifying geographic disparities, catalyzing multisector actions, and evaluating the effects of interventions designed to improve population health and advance equity. Lessons learned and user-friendly resources are provided to help accelerate widespread scaling of these efforts.
